# Supplementary material for: Model-based test case prioritization using selective and even-spread count-based methods with scrutinized ordering criterion
Source: PLoS One. 2020 Feb 21;15(2):e0229312. doi: 10.1371/journal.pone.0229312 (PMC7034818; doi:10.1371/journal.pone.0229312)
Supplement: Attachment — Submitted filename: Table of Correction (PLOS ONE).docx [file pone.0229312.s001.docx]

TABLE OF CORRECTIONS

(RESPONSES TO EDITOR’S & REVIEWER’S COMMENTS)

| **No** | **Comment** | **Reply to Comment** | **For manuscript with NO track changes** | |
| --- | --- | --- | --- | --- |
|  |  |  | **Prev Page #**  **(Line #)** | **New Page #**  **(Line #)** |
| Journal Requirements | | | | |
| 1. | We note you have included a table to which you do not refer in the text of your manuscript. Please ensure that you refer to Table 2 in your text; if accepted, production will need this reference to link the reader to the Table. | Table 2 has already been referred in the text. | 35  (809) | 29  (674) |
| Academic Editor | | | | |
| 2. | One of my observation is the problem statement and emphasis on regression testing execution costs, and the claim that 80% of the testing costs are spent on reg. testing execution. With modern unit test frameworks, the execution cost of regression testing doesn’t seem to be an issue anymore as the framework simply re-runs the predefined tests. It’s better that the authors further elaborate/revise the problem statement. | Added supporting point in the problem statement regarding regression testing execution costs issue. | - | 3  (43) |
| 3. | There are some inconsistencies between the reference styles used. For example, page 3 last paragraph, “Yoo and Harman (1)…” while others use []. Please fix this issue. | Fixed the reference style in Endnote. | 3  (60) | 3  (56) |
| 4. | Please improve the English. | Done proofread the manuscript. |  |  |
| 5. | How does the proposed approach compared with machine learning based test case generation/prioritizations technique? It would be good if the paper addresses this. | The suggestion is considered by mentioning it in the recommendations for future work. | - | 42  (916) |
| 6. | How does the proposed approach address the oracle problem? | Added the explanation how the proposed approach addresses the oracle problem. The explanation is placed in section 2.1 because how the proposed approach addresses the oracle problem is based on how MBT addresses it. | - | 6  (131) |
| 7. | I’m not sure of whether the authors have made all the data available, or provided enough detail for another researcher to reproduce the findings. Can you please clarify? | Uploaded all the relevant data to replicate the experiment in the public repository, https://doi.org/10.7910/DVN/5LZ51B. Added the reference in text. Also added statements and citations in the text about where the data are used. | - | 23(542),  27(621),  28(641),  28(657),  29(676),  34(751) |
| 8. | I noted that the validation of the method presented in this manuscript might be incomplete. Whilst the performance has been compared to other existing methods, I’m unsure whether the methods chosen for comparison are state-of-the-art, and thus the results may not have been put in the right context. Please clarify this too. | Based on our systematic literature review (reference in text), the selected existing approaches are among the latest ones that are replicable for our experiment. There are other more latest approaches but they used different models. We had explained in the text why we only selected approaches that used FSM model and not other models. | 23  (541) | 24  (549) |
| 9. | The structure of the paper is not adequate for a research paper. It seems the paper is written in a way a thesis should be written, especially section 5. I advise the authors to restructure the paper in a manner that is more acceptable for research papers rather than a thesis such as, Abstract, Introduction, Related work, Method, Experiments, Results, Discussion, Conclusion. | Restructured the paper according to the recommendation. | Section 5 | Section 5,6,7 |

| Reviewer 1 | | | | |
| --- | --- | --- | --- | --- |
| 10. | Introduction section is very lengthy. Some information pertaining to the research is better to be discussed in section 4. | Shortened the introduction content. | Section 1 | Section 1 |
| 11. | In am not sure what is the different between section 2 and 3. This is because both sections has similar contents. The background section should be more general, while the related work should cover the works that are applied in the current research | Section 2 and 3 are actually different in terms of their contents. In section 2, first we explained what is MBT and its process in general. This explanation is essential because the experiment to evaluate the MB-TCP approaches in this study follows the MBT steps. Then, a brief introduction to FSM model is presented because this model is utilized in our MB-TCP approach and the benchmark approaches. Lastly, we introduced about MB-TCP. We started from TCP in general and then narrowed our explanation into MB-TCP. In MB-TCP sub section, we also included a sub section that explained about MB-TCP using FSM model. We considered section 2 as general because no specific work regarding MB-TCP approach has been discussed in detail. Section 3, on the other hand, did discussed MB-TCP approaches in detail. In section 3, we included several existing MB-TCP approaches in the literature, elaborated their proposed approaches and identified their limitations. | Section 2,3 | Section 2,3 |
| 12. | The heading of section 4 is proposed MB-TCP approach. It is confusing as prior to this, it was mentioned in section 3 that MB-TCP approach is an existing approach. To reduce the confusion, heading for section 4 is better as “proposed approach” | Changed. | 16  (363) | 15  (353) |
| 13. | The authors did not explain why they have selected the three websites only. Justification needed. | Added explanation why the three web apps are chosen. | - | 23  (527) |
| 14. | The main issue that forms the basis for research is the high execution cost. However, the authors did not perform testing of their proposed method on the execution cost. This is the biggest limitation of this research. We cannot determine the effectiveness of the proposed approach in relation to the execution cost as it is major issue highlighted in the abstract. | We did not emphasized regarding execution cost during the experimentation because the scope was only in terms of approaches’ effectiveness (not efficiency) in prioritizing faults detecting tests, as can be observed by the evaluation metric (APFD) that we used.  Nonetheless, we acknowledged that the execution cost of prioritization approaches is also important to be measured. Therefore, for this comment, we have added another recommendation for future work that we will also evaluate the approaches regarding their execution cost. | - | 42  (917) |
| 15. | The sample size is based on the number of websites. However, each website has different line of command. I think it is better to use line of code as research unit rather than the website itself. | APFD value is the dependent variable which we want to observe in this experiment. The APFD value is calculated from the ordering of test cases in a test suite. This ordering of test cases is generated from the prioritization approaches. Here, the prioritization approaches act as the independent variable because they affect the APFD value that is generated. Also, this means that from one test suite, we can get one APFD value, which constitute one sample. In this experiment, there are three test suites and they are produced from the three websites. Therefore, the sample size is three. That is why we considered the number of websites as the sample size. Because the number of websites will determine how many test suites there are, how many APFD values will be generated and how many samples there will be. Line of code as the sample size is not possible because it does not dictate how many APFD values to be generated. | 29  (677) | 31  (696) |
| 16. | The use of Shapiro–Wilk test for hypothesis testing is questionable. The null hypothesis is that there are no differences between the APFD values from the existing MB-TCP approaches using FSM and SESOC. However, Shapiro–Wilk test is to test the normality. The finding suggests that that SESOC is not normally distributed. It did not suggest that they are different. The authors should use ANOVA as described in section 4. | We used Shapiro–Wilk test to check whether the dependent variable is approximately normally distributed for each category of the independent variable. From the test, it is shown that the dependent variable for SESOC is not normally distributed as tabulated in Table 9. That is why we did not used ANOVA. Consequently, we used the Kruskal-Wallis H test to test whether there are differences between the APFD values from the existing MB-TCP approaches using FSM and SESOC. | 41  (911) | 37  (815) |
| 17. | Figures are off poor quality and cannot be comprehended. Some figures are missing from the document. | Improved the figures quality according to the journal’s requirements. | - | - |
